# Antioxidant and Biological Activities of *Acacia saligna* and *Lawsonia inermis* Natural Populations

**DOI:** 10.3390/plants9070908

**Published:** 2020-07-17

**Authors:** Hosam O. Elansary, Agnieszka Szopa, Paweł Kubica, Halina Ekiert, Fahed A. Al-Mana, Mohammed A. Al-Yafrsi

**Affiliations:** 1 Plant Production Department, College of Food and Agricultural Sciences, King Saud University, P.O. Box 2455, Riyadh 11451, Saudi Arabia; falmana@ksu.edu.sa (F.A.A.-M.); malyafrsi@ksu.edu.sa (M.A.A.-Y.); 2 Floriculture, Ornamental Horticulture, and Garden Design Department, Faculty of Agriculture (El-Shatby), Alexandria University, Alexandria 21545, Egypt; 3 Department of Geography, Environmental Management, and Energy Studies, University of Johannesburg, APK Campus, Johannesburg 2006, South Africa; 4 Department of Pharmaceutical Botany, Medical College, Jagiellonian University, ul. Medyczna 9, 30-688 Kraków, Poland; p.kubica@uj.edu.pl (P.K.); halina.ekiert@uj.edu.pl (H.E.)

**Keywords:** *Acacia saligna*, *Lawsonia inermis*, polyphenols, antioxidant, antiproliferative, cytotoxicity, antibacterial, antifungal

## Abstract

*Acacia saligna* and *Lawsonia inermis* natural populations growing in Northern Saudi Arabia might be a valuable source of polyphenols with potent biological activities. Using high-performance liquid chromatography–diode array detection (HPLC-DAD), several polyphenols were detected tentatively in considerable amounts in the methanolic leaf extracts of *A. saligna* and *L. inermis. A. saligna* mainly contained rutoside, hyperoside, quercetin 3-glucuronide, gallic acid and *p*-coumaric acid, whereas those of *L. inermis* contained apigenin 5-glucoside, apigetrin and gallic acid. Strong antioxidant activities were found in the leaf extracts of both species due to the presence of hyperoside, quercetin 3-glucuronide, gallic acid, isoquercetin, *p*-coumaric acid, quercitrin and rutoside. *A. saligna* and *L. inermis* leaf extracts as well as hyperoside, apigenin 5-glucoside, and quercetin 3-glucuronide significantly reduced reactive oxygen species accumulation in all investigated cancer cells compared to the control. Methanolic leaf extracts and identified polyphenols showed antiproliferative and cytotoxic activities against cancer cells, which may be attributed to necrotic cell accumulation during apoptotic periods. Antibacterial activities were also found in both species leaf extracts and were twice as high in *A. saligna* than *L. inermis* due to the high composition of rutoside and other polyphenols. Finally, strong antifungal activities were detected, which were associated with specific phenols such as rutoside, hyperoside, apigenin 5-glucoside and *p*-coumaric acid. This is the first study exploring the polyphenolic composition of *A. saligna* and *L. inermis* natural populations in northern Saudi Arabia and aiming at the detection of their biological activities.

## 1. Introduction

Natural populations of medicinal plants are considered as important sources of natural compounds that may have bio/pharmacological activities. Large portions of the global population are reliant on these botanical remedies as traditional or alternative medicine, while others consume or utilize these plants and their products as nutritional supplements [1]. Natural compounds such as polyphenols display interesting medicinal properties. These polyphenols may reduce certain age-associated diseases such as cancer and Alzheimer’s disease by mitigating increased cellular damage through the reduction of reactive oxygen species (ROS) by the down-regulation of antioxidant enzymes [2,3] and the modulation of the inflammatory status, autophagy function and gut microbiota [4]. The antiproliferative and cytotoxic effects of polyphenols on cancer cells are attributed to cell cycle arrest and the molecular regulation of specific genes [5,6,7,8,9]. Polyphenols may control microbes such as bacteria and fungi [10,11] and might be used as natural food preservatives [12]. Medicinal and aromatic plant polyphenols available in different parts of the plant (leaves, stems, flowers, seeds and bark) were successful in controlling foodborne pathogens such as *Escherichia coli*, *Staphylococcus aureus*, and *Bacillus cereus*; human pathogenic fungi such as *Candida albicans*; and plant pathogenic molds such as *Penicillium funiculosum* [6,13,14].

*Acacia* (wattle) are woody species belonging to the pea family (Fabaceae), and this genus comprises over 1350 species that are distributed in the warm regions of the world, including Australia, the Americas, Africa and Asia [15]. *Acacia saligna* (Labill.) Wendl is an invasive, fast-growing woody tree in Saudi Arabia that produces seeds that have a considerable amount of protein (18.25% to 35.5%) [15]. However, the polyphenol profile of *A. saligna* is quite unknown. Previous investigations have shown that Egyptian *A. saligna* leaf extracts may qualitatively contain flavonoids—e.g., quercetin, quercitrin, apigenin, apigenin 7-glucoside, astragalin, luteolin, myricetin, myricitrin and kaempferol, and phenolic acids—e.g., gallic acid, and catechins such as catechin and 7-galloylcatechin [16,17]. Another study on the flower’s extracts detected flavonoids such as quercetin, naringenin and kaempferol and phenolic acids such as benzoic acid, caffeic acid, *o*-coumaric acid, *p*-hydroxybenzoic acid and ellagic acid [18].

Few studies have investigated the biological activities of the flower and leaf extracts of *Acacia* species, particularly *A. saligna*. Egyptian *A. saligna* flower water extracts showed weak antioxidant and antibacterial activities but good antifungal activity [18]. Egyptian *Acacia nilotica* and *Acacia seyal* leaf extracts showed higher antioxidant activities than *Acacia laeta* extracts [19]. A study on the aerial parts of *Acacia* species *(A. salicina*, *A. laeta*, *A. hamulosa*, and *A. tortilis)* from an eastern region of Saudi Arabia (approximately 700–1000 km from the Riyadh region) revealed that *A. laeta* and *A. hamulosa* have cytotoxic activities against HepG2 and breast cancer cell line [20]. Other *Acacia* species flower extracts showed allelopathic effects against *Hordeum murinum* [21]. However, to our best knowledge, this is the first investigation into the antioxidant, cytotoxic and antimicrobial activities of Saudi natural populations of *A. saligna*.

*Lawsonia inermis* (henna tree or Egyptian privet) is the natural source of the dye henna which is commonly used to dye skin and hair worldwide. The plant belongs to the family Lythraceae and is native to Africa, Asia and northern Australia. The leaves are used traditionally for the preparation of henna dye. The natural constituents of *L. inermis* are lawsone (2-hydroxy-1,4-naphthoquinone), essential oil, tannins, terpenoids, lipids, coumarins, flavonoids: linarigenin (4′-methoxyapigenin), apigenin 7-glucoside, apigenin-glucoside, luteolin, luteolin 7-glucoside, cosmosiin, and phenolic acids: gallic acid and *p*-coumaric acid [22]. Previous investigations on Tunisian *L. inermis* revealed that the butanolic fraction of leaves have strong antioxidant activities, and these activities were attributed to phenolic glycosides including 1,2,4-trihydroxynaphthalene-1-O-β-d-glucopyranoside [23]. In another study, the Tunisian *L. inermis* leaf and seed aqueous extracts showed antioxidant activities, but no active compounds were identified [24]. Iranian *L. inermis* aqueous leaf extracts showed diverse antioxidant activities associated with ecotypes [25]. Indian *L. inermis* had significant antioxidant activities that inhibited Cr (VI)-induced cytotoxicity in MDA-MB-435S breast cancer cells [26]. No antioxidant, cytotoxic or antimicrobial studies were conducted on Saudi *L. inermis* natural populations. These populations may have unique phytochemical profiles and could be considered as rich sources of secondary metabolites.

In the current study, the polyphenolic composition and biological effects of natural populations of *A. saligna* and *L. inermis* growing in the northern region of Saudi Arabia were explored. The polyphenol compounds were tentatively estimated qualitatively and quantitatively using the high-performance liquid chromatography–diode array detection (HPLC-DAD) method. The antioxidant activities of the methanolic extracts and identified polyphenols were investigated by three methods. The antiproliferative and cytotoxic activities were studied against different human cancer cells. The antibacterial and antifungal activities of leaf extracts and identified polyphenols were examined for the first time against a wide spectrum of microorganisms.

## 2. Materials and Methods

### 2.1. Plant Material and Preparation

Leaves of *Acacia saligna* (Labill.) Wendl and *Lawsonia inermis* L. were obtained from natural populations growing in the northern Riyadh region, Saudi Arabia. Plants were identified and vouchered at the College of Food and Agricultural Sciences, King Saud University, Riyadh (Hosam0002217–106) by Hosam Elansary. To obtain leaf extracts, fresh leaves were lyophilized, powdered and then extracted with methanol (0.5 g dry weight (DW)) in 10 mL twice by sonication for 30 min at 30 °C. Purification was conducted using Whatman paper; then, the residues were dried at 25 °C (to eliminate the methanol) then frozen at −80 °C. For HPLC study, the residues were dissolved in methanol (1 mL; Merck, Kenilworth, NJ, USA), whereas for bioassays, methanol was totally removed using a rotary evaporator [27]. All experiments were approved by the College of Agriculture, Alexandria University, Egypt (2018–2020–4275). Bacteria, fungi and cancer cell lines (American Type Culture Collection) were obtained from the Faculty of Agriculture, Alexandria, Egypt.

### 2.2. Analyses of Phenolic Compounds

The qualitative and quantitative estimations were performed by high-pressure liquid chromatography (HPLC) analysis using a Merck-Hitachi liquid chromatograph (LaChrom Elite, Berlin, Germany) with an L-2455 diode array detector (DAD). The used analytical column was Purospher RP-18e solid phase column (250 × 4 mm; 5 μm, Merck, Kenilworth, NJ, USA). As the eluent for the gradient program, we used A—methanol and B—a 0.5% acetic acid and methanol mixture 1:4 (*v*/*v*). The solvent ratio changed over time as follows: 0–20 min, 100% B; 20–35 min, 100–80% B; 35–55 min, 80–60% B; 55–70 min, 60–0% B; 70–75 min, 75–90 min, 100% B. The flow rate was 1 mL/min and the sample injection volume was 10 µL. Temperature was maintained at 25 °C. The DAD analytical waveband ranged from 200 to 400 nm. Quantitative analysis was conducted at 254 nm. The HPLC validation was carried out earlier [28,29]. Compounds contained in extracts were identified by comparing their retention times and UV spectra with standards. Quantitative measurements were conducted using calibration curves (Figure S1).

For the HPLC analysis, 46 standard substances were used (Sigma-Aldrich, Berin, Germany) from two chemical groups: phenolic acids and their derivatives (25 compounds)—bensoic acid, 3-phenylacetic acid, caffeic acid, caftaric acid, chlorogenic acid, cinnamic acid, *m*-coumaric acid, *o*-coumaric acid, *p*-coumaric acid (y = 31,143,463.3x + 37,623.6, R^2^ = 0.999), cryptochlorogenic acid, 3,4-dihydroxyphenylacetic acid, elagic acid, ferulic acid, gallic acid (y = 71,108,023.2x + 11,440.5, R^2^ = 0.999), gentisic acid, *p*-hydroxybenzoic acid, hydroxycaffeic acid, isochlorogenic acid, neochlorogenic acid, phenylacetic acid, protocatechuic acid, rosmarinic acid, salicylic acid, syringic acid and vanillic acid—as well as flavonoids (21 compounds)—apigenin (y = 59,476,721.1x − 1,168,407.0, R^2^ = 0.999), apigenin 5-glucoside (y = 38,556,890.2x − 563,218.5, R^2^ = 0.999), apigetrin, cynaroside, hyperoside (y = 83,592,227.7x − 69.9, R^2^ = 0.998), isoquercetin (y = 88,387,200.2x + 113,000.0, R^2^ = 0.999), kaempferol, kaempferol-7 rhamnoside, luteolin, myricetin, naringin, populin, quercetin (y = 67,269,930.4x − 813,339.4, R^2^ = 0.999), quercetin 3-glucuronide (y = 81,939,087.5x − 794,095.4, R^2^ = 0.999), quercimetrin, quercitrin, rhamnetin, robinin, rutoside (y = 59,420,774.0x + 66,560.0, R^2^ = 0.999), trifolin and vitexin.

### 2.3. Antioxidant Activity

The antioxidant activities of leaf extracts of *A. saligna* and *L. inermis* were determined using ferric reducing antioxidant power (FRAP), β-carotene bleaching and 2,2-diphenyl-1-picrylhydrazyl (DPPH) assays [30,31,32,33,34]. The amount of leaf extracts required to scavenge 50% of β-carotene bleaching/DPPH solution/FRAP reagent was defined as the IC_50_ (µg/mL) and was determined by plotting the inhibition percent against extract concentration.

In the DPPH experiments, 5 mL of 0.004% methanolic DPPH solution was added to methanolic leaf extracts serial concentrations, then incubated for 30 min at room temperature in the dark. Finally, the absorbance was measured at 517 nm. The DPPH-free radical inhibition was determined as follows:

The percentage inhibition of antioxidant activity (IAA) was calculated in triplicate:

(1) $$\mathrm{IAA}\;=\;\;\frac{{\left(AB_{5170nm}\right)}_{C}-{\left(AB_{517nm}\right)}_{s}}{{\left(AB_{517nm}\right)}_{C}}\times 100$$

where ${\left(AB_{5170nm}\right)}_{C}$ and ${\left(AB_{517nm}\right)}_{s}$ are Abs.517 nm of the control and sample, respectively.

The inhibition concentration of each sample was compared with that of the BHT (butylated hydroxytoluene) as a positive control and blank.

In the β-carotene-bleaching assay, the mixture was prepared by dissolving the β-carotene (0.5 mg) in chloroform (1 mL), then adding linoleic acid (25 μL) and Tween 40 (200 mg). A vacuum evaporation was used to remove the chloroform; finally, the distilled water was added (100 mL) and followed by vigorous shaking. The mixture (2.5 mL) was added to serial concentrations of leaf extracts, then incubated for 48 h at room temperature, and the absorbance was measured at 470 nm.

In the FRAP assay, aliquots (100 μL) of leaf extracts/Trolox (Sigma-Aldrich, Berlin, Germany) were added to the prepared FRAP reagent (3 mL), then mixed vigorously and incubated for 30 min at 37 °C. The calibration procedure of FRAP was conducted by applying serial dilutions of Trolox (0–0.5 mmol/L), as standard. The absorbance was determined at 593 nm for FRAP. All antioxidant experiments were conducted in triplicates and repeated thrice.

### 2.4. Detection Intercellular ROS Accumulation

To assay the capacity of *A. saligna* and *L. inermis* methanolic extracts as well as identified polyphenols to reduce the intracellular levels of ROS in HeLa, Jurkat, T24, and MCF-7 cancer cells, the fluorogenic dye H_2_DCF-DA was used. Following passive diffusion into the cells, H_2_DCFDA was deacetylated using esterases into the nonfluorescent compound. That compound was oxidized by ROS and converted to the highly fluorescent 2′,7′-dichlorofluorescein (DCF) [35]. Cells were grown in 96-well plates at a density of 1 × 10^4^ cells per well for 24 h before experiments; then, the culture medium was changed on 10 μM H_2_DCFDA (Sigma Aldrich, St. Louis, MO, USA) in serum-free medium (MEM) and incubated for 45 min before applying treatments. Then, cancer cells were exposed to plant extracts and identified polyphenol DPPH IC_50_ values. Cells treated with 1 mM hydrogen peroxide (H_2_O_2_) were considered as a positive control. The DCF fluorescence was measured after 90 min using a microplate reader FilterMax F5 (Thermo Fisher Scientific, Waltham, MA, USA) at 485 nm.

### 2.5. Anticancer Activities

Antiproliferative and cytotoxic effects of the leaf extracts of *A. saligna* and *L.* were tested against HeLa, HT-29, Jurkat, MCF-7 and HEK-293 (normal human cells) [5,13,31,36]. To study the antiproliferative effects on cell viability, a 3-(4,5-dimethylthiazol-2-yl)-2,5-diphenyltetrazolium bromide (MTT) assay was used. Leaf extracts were solubilized in DMSO (1%) then added to standard media (MEM) containing (10% FBS, 0.1 mM non-essential amino acids, 17.8 mM NaHCO_3_, and 1 mM sodium pyruvate) and cancer cells in 75 cm^2^ flasks. The cancer cells were prepared in microtiter plates at 4 × 10^−4^ cells per µL in 270 µL medium for 48 h (37 °C, 5% CO_2_). Leaf extract serial concentrations were used until final concentrations of 50, 100, 200, 300 and 400 µg/mL were reached. Then, a washing procedure was performed using PBS. The MTT was dissolved in PBS then added (12 mM) to the medium. Finally, isopropanol (0.04 N HCl) was mixed with the MTT solution, and the mixture was incubated at room temperature for 40 min. A negative control (untreated) and positive couple (vinblastine sulfate and taxol) were used. To calculate the inhibition activity percentage (IAA) obtained from measuring the absorbance at a 570 nm wavelength, the following equation was used:

(2) $$IAA=\frac{{\left(AB_{570nm}\right)}_{C}-{\left(AB_{570nm}\right)}_{s}}{{\left(AB_{570nm}\right)}_{C}}\times 100$$

where *AB* is the absorbance and ${\left(AB_{570nm}\right)}_{C}$ and ${\left(AB_{570nm}\right)}_{s}$ are Abs.570 nm of the control and sample, respectively.

The percentage of viable cells was plotted against the extract concentration (µg mL^−1^) to determine the IC_50_. The IC_50_ amounts were applied in the flow cytometry assay to study the cytotoxic activities of *A. saligna* and *L. inermis* leaf extracts; then, the apoptotic cell populations were determined (FACScan, New York, NY, USA) [5,13,36,37]. The assay was based on monitoring phosphatidylserine translocation in the Annexin [38]. In viable cells, the phosphatidylserine is located in the inner layer of the plasma membrane. Early apoptosis starts when the phosphatidylserine translocates from the inner to the outer layer of the plasma membrane, and apoptotic cells are reflected by measuring the binding of Annexin V-FITC to external phosphatidylserine. The flow cytometer data were presented in quadrants as percentages: lower left, viable cells; upper left, necrotic cells; lower right, early apoptotic cells; and upper right, late apoptotic cells.

### 2.6. Antibacterial Effect

Bacterial isolates of *Listeria monocytogenes* (clinical isolate), *Escherichia coli* (ATCC 35210), *Staphylococcus aureus* (ATCC 6538), *Bacillus cereus* (ATCC 14579), *Micrococcus flavus* (ATCC 10240) and *Pseudomonas aeruginosa* (ATCC 27853) were used in this experiment. A microtiter plate-based protocol (micro-dilution) was used following the procedure in [14,39,40,41], by preparing serial concentrations of *A. saligna* and *L. inermis* extracts that were mixed with bacterial inoculum at 1.0 × 10^4^ CFU in each well with 100 μL tryptic soy broth per well, then incubated for one day at 37 °C in a rotary shaker. The minimum inhibitory concentration (MIC) was defined as the lowest concentration that caused no visible growth by a binocular microscope. The minimum bactericidal concentration (MBC) was determined using the serial subculturing of extracts (2 μL), and the minimal concentration causing the elimination of 99.5% of each inoculum was considered as the MBC value. The optical density (OD) was determined using a 655 nm wavelength. A positive control (streptomycin) at 0.01–10 mg/mL was used alongside the negative one (DMSO, 1%).

### 2.7. Antifungal Effect

*A. saligna* and *L. inermis* antifungal effects were determined against *P. ochrochloron* (ATCC 48663), *A. ochraceus* (ATCC 12066), *C. albicans* (ATCC 12066), *A. niger* (ATCC 6275), *A. flavus* (ATCC 9643) and *P. funiculosum* (ATCC 56755) using a micro-dilution method [37,39,40]. The MIC was determined by a stereomicroscope. The minimum fungicidal concentration (MFC) was determined by preparing serial dilutions of 2 µL extracts in sub-cultures of fungi at 28 °C for 72 h in microtiter plates that contained 100 µL of broth medium. Ketoconazole (KTZ, 1–3500 µg/mL) was used as a positive control; DMSO (1%) was also used.

## 3. Results

### 3.1. A. saligna and L. inermis Polyphenol Profiling of Leaf Extracts

**Figure 1:**
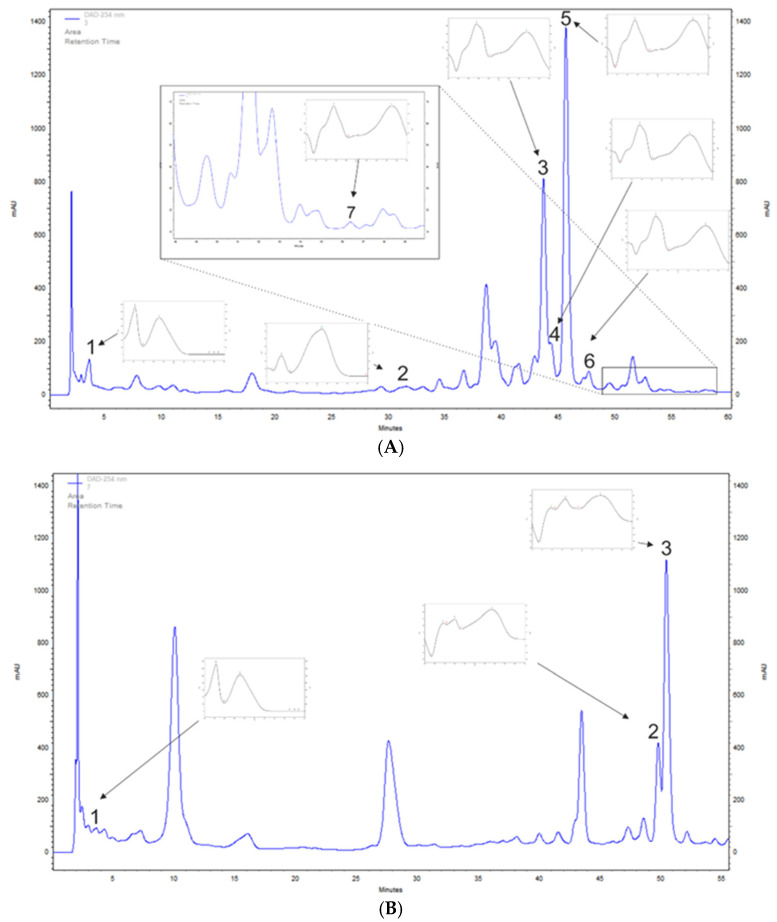
Examples of high-performance liquid chromatography–diode array detection (HPLC-DAD)-based separation (λ = 254 nm) of leaf extracts of (**A**) *Acacia saligna*: 1, gallic acid; 2, *p*-coumaric acid; 3, hyperoside; 4, quercetin 3-glucuronide; 5, rutoside; 6, isoquercetin; 7, quercetin; and (**B**) *Lawsonia inermis:* 1, gallic acid; 2, apigenin 5-glucoside; 3, apigetrin.

*A. saligna* and *L. inermis* methanolic leaf extracts polyphenols were determined tentatively with the HPLC-DAD method to detect chosen phenolic acids and flavonoids. *A. saligna* methanolic leaf extracts contained phenolic acids such as gallic acid, at 136.2 mg/100 g DW, and *p*-coumaric acid, at 34.8 mg/100 g DW. High amounts of flavonoid were detected including rutoside, at 1533.0 mg/100 g DW; hyperoside (quercetin 3-galactoside), at 632.5 mg/100 g DW; quercetin 3-glucuronide, at 125.5 mg/100 g DW; isoquercetin, at 73.2 mg/100 g DW; and quercetin, at 6.1 mg/100 g DW (Table 1 and Figure 1A).

*L. inermis* leaf extracts contained high amounts of apigetrin, at 1180.9 mg/100 g DW; apigenin 5-glucoside, at 596.3 mg/100 g DW; and gallic acid, at 81.0 mg/100 g DW (Table 1 and Figure 1B).

### 3.2. Antioxidant Effects

**Figure 2:**
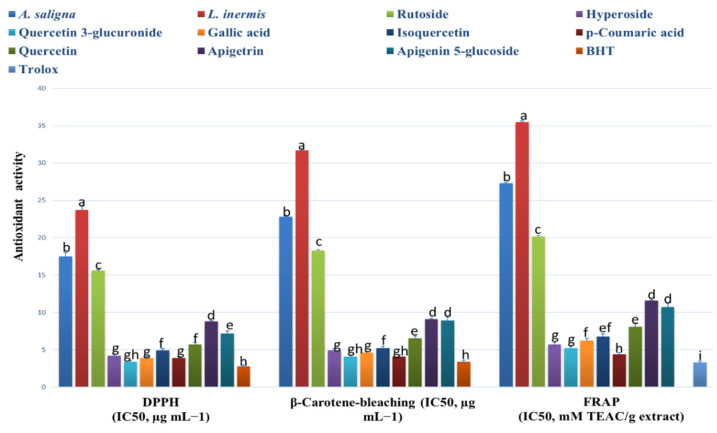
*A. saligna* and *L. inermis* antioxidant effects using 2,2-diphenyl-1-picrylhydrazyl (DPPH), β-Carotene bleaching and ferric reducing antioxidant power (FRAP) assays (expressed as IC_50_ in µg/mL). Identified polyphenol (rutoside, hyperoside, quercetin 3-glucuronide, gallic acid, isoquercetin, *p*-coumaric acid, quercitrin, apigetrin and apigenin 5-glucoside) antioxidant effects were also added. Values are expressed as mean ± standard deviation. TEAC: Trolox equivalent antioxidant capacity. Different letters among group of columns indicate significant differences (*p* ≤ 0.05).

*A. saligna* and *L. inermis* methanolic extracts showed antioxidant activities comparable to the identified polyphenols, as shown in Figure 2. *A. saligna* showed significantly higher antioxidant activity than *L. inermis* as measured by different assays (DPPH, B-carotene bleaching and FRAP). In *A. saligna*, the identified polyphenols including hyperoside, quercetin 3-glucuronide, gallic acid, isoquercetin, *p*-coumaric acid and quercitrin showed strong antioxidant activities, with quercetin 3-glucuronide having the lowest IC_50_ value. In *L. inermis*, identified polyphenols including apigetrin and apigenin 5-glucoside showed strong antioxidant effects. Quercetin 3-glucuronide, gallic acid and *p*-coumaric acid antioxidant activities were comparable to antioxidant standards.

### 3.3. MTT Assay and Flow Cytometry

**Figure 3:**
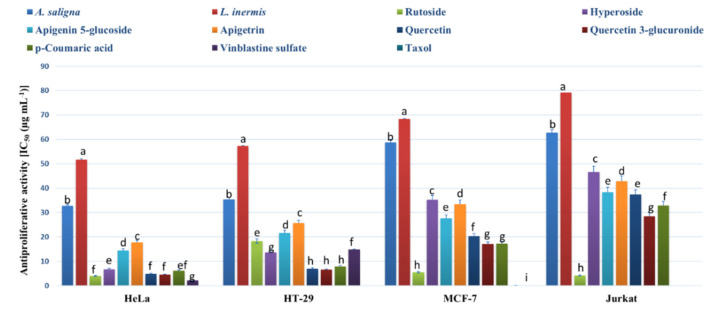
Antiproliferative activity (IC_50_ (µg mL^−1^)) of *A. saligna* and *L. inermis* methanolic extracts, rutoside, hyperoside, apigenin 5-glucoside, apigetrin, quercetin and *p*-coumaric acid against cancer cells: cervical adenocarcinoma (HeLa), T-cell lymphoblast like (Jurkat), breast adenocarcinoma cultures (MCF-7), colon adenocarcinoma (HT-29) and HEK-293 (normal human cells). HEK-293 was not presented in the figure and showed IC_50_ values > 400 µg mL^−1^ for all extracts and polyphenols. Different letters among group of columns indicate significant differences (*p* ≤ 0.05).

The MTT assay was employed to determine the antiproliferative activities of methanolic leaf extracts and the identified polyphenols against an array of cancer cells (Figure 3). Strong antiproliferative effects were exhibited by *A. saligna* and *L. inermis* leaf extracts as well as the identified polyphenols against all cells except the normal cells of HEK-293 (showing IC_50_ values > 400 µg mL^−1^). *A. saligna* showed significantly higher antiproliferative activities than *L. inermis*. Strong antiproliferative activities were detected when using identified polyphenols including rutoside, hyperoside, quercetin, quercetin 3-glucuronide and *p*-coumaric acid against cancer cells.

**Figure 4:**
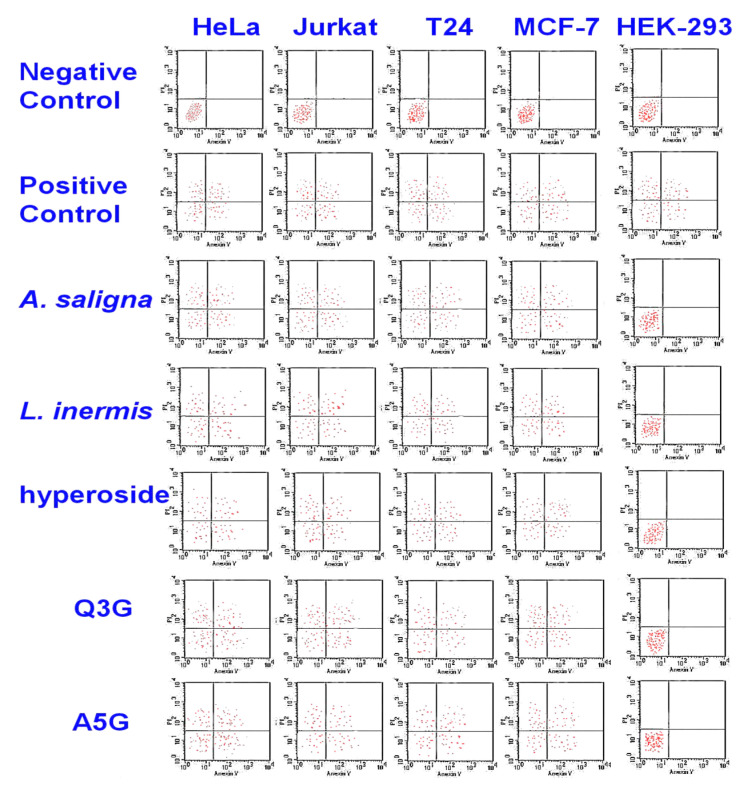
Cytotoxicity of *A. saligna* and *L. inermis* methanolic extracts, hyperoside, quercetin 3-glucuronide (Q3G) and apigenin 5-glucoside (A5G), as estimated with flow cytometry. There was an accumulation of early apoptotic cells in the lower-right quadrant as well as accumulation of the late apoptotic cell in the upper-right quadrant in treated cancer cells.

The cytotoxic activities of *A. saligna* and *L. inermis* leaf extracts as well as hyperoside, apigenin 5-glucoside and quercetin 3-glucuronide were investigated by monitoring phosphatidylserine translocation in the annexin assay (Figure 4). In viable cells, the phosphatidylserine is located in the inner layer of the plasma membrane. Early apoptosis starts when the phosphatidylserine translocates from the inner to the outer layer of the plasma membrane, and apoptotic cells are reflected by measuring the binding of Annexin V-FITC to external phosphatidylserine. The flow cytometry showed the apoptotic cell accumulation following 48 h of exposure in the upper and lower-right quadrant. The cytotoxic activities of *A. saligna* and *L. inermis* leaf extracts as well as major identified polyphenols were confirmed. The cell death was induced by the leaf extracts hyperoside, quercetin 3-glucuronide (Q3G) and apigenin 5-glucoside (A5G).

### 3.4. ROS Accumulation

**Figure 5:**
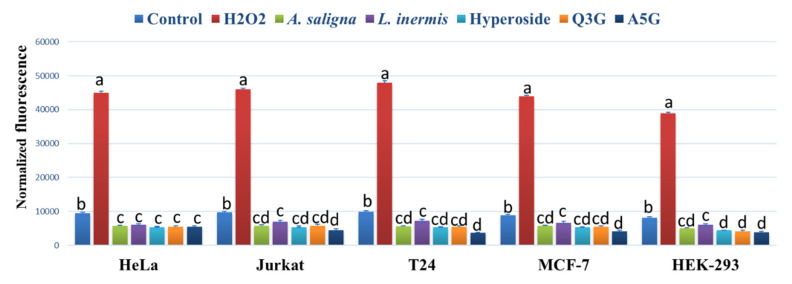
The effect of *A. saligna* and *L. inermis* methanolic extracts, hyperoside, quercetin 3-glucuronide (Q3G), and apigenin 5-glucoside (A5G) DPPH IC_50_ on the 2′,7′-dichlorofluorescein (DCF) fluorescence in HeLa, Jurkat, T24, MCF-7 and HEK-293 cells. A medium with 1 mM hydrogen peroxide (H_2_O_2_) was used as a positive control. The data are expressed as the mean ± SD of three experiments (four replicates per treatment). Different letters among group of columns indicate significant differences (*p ≤ 0.05*).

*A. saligna* and *L. inermis* leaf extracts as well as hyperoside, apigenin 5-glucoside and quercetin 3-glucuronide significantly reduced the ROS accumulation in all investigated cancer cells compared to controls using the intercelleular ROS accumulation assay of H_2_DCFDA fluorescence (Figure 5). The highest reduction of ROS was achieved using apigenin 5-glucoside (A5G) in Jurkat, T24 and MCF-7 following 90 min of incubation. H_2_O_2_ showed the highest levels of ROS accumulation in all cancer cells.

### 3.5. Antibacterial Activities of A. saligna and L. inermis Leaf Extracts

*A. saligna* and *L. inermis* extracts showed antibacterial effects against different bacteria (Table 2)*. A. saligna* showed two-fold higher antibacterial activities than *L. inermis* against all bacteria. The most sensitive bacteria were *E. coli* and *S. aureus* (showing the lowest IC_50_ values), while the most resistant were *L. monocytogenes* and *M. flavus*. Identified polyphenols including rutoside, apigenin 5-glucoside and *p*-coumaric acid showed strong antibacterial activities against all bacteria except hyperoside, quercetin and quercetin 3-glucuronide, showing moderate to low antibacterial effects.

### 3.6. Antifungal Effects of Leaf Extracts

*A. saligna* and *L. inermis* extracts showed strong antifungal effects against most fungi studied (Table 3). *A. saligna* showed two to three-fold higher activity (lower IC_50_ values) than *L. inermis.* Rutoside, hyperoside, apigenin 5-glucoside and *p*-coumaric acid showed the highest antifungal effects among the studied polyphenols.

## 4. Discussion

Few studies have investigated the phenolic acid and flavonoid composition of *A. saligna* and *L. inermis* leaf extracts. This is the first study to quantitatively determine compounds from phenolic acids and flavonoids in Saudi *A. saligna* and *L. inermis* natural populations (Table 1). The main polyphenols were rutoside (1533.0 mg/100 g DW), hyperoside (quercetin 3-galactoside) (632.5 mg/100 g DW), quercetin 3-glucuronide (125.5 mg/100 g DW), isoquercetin (73.2 mg/100 g DW) and quercetin (6.1 mg/100 g DW). In *A. saligna* leaf extracts, high amounts of gallic acid (136.2 q) and *p*-coumaric acid (34.8 mg/100 g DW) were also confirmed for the first time in the context of Arabian-origin plants. El Sissi et al. [16] isolated the flavonoids: quercitrin, astragalin, myricitrin and kaempferol as well as quercetin from the leaves of *A. saligna* growing in Cairo (Egypt), which were found also in our samples. El-Toumy et al. [17] studied the ethyl-acetate extract of of *A. saligna* leaves harvested from Cairo (Egypt) for phenolic compound detection. They confirmed structures similar to our Arabian origin samples: gallic acid, quercetin, quercetin 3-arabinoside, quercetin 3-rhamnoside, and additionally: apigenin, apigenin 7-glucoside, catechin, 7-galloylcatechin, myricetin 3-rhamnoside, myricetin 3-arabinoside, myricetin 3-glucopyranoside, luteolin and myricetin. *A. saligna* leaf extracts cultivated in Damietta City (Egypt) were studied by Sahar et al. [42] for pirostane saponin as well as biflavonoid glycoside. They detected some flavonoids including myricetin 3-rhamnoside, erythrodiol and quercetin 3-rhamnoside. Recently, Al-Huqail et al. [18] studied the Egyptian *A. saligna* flower extracts for antifungal, antibacterial and antioxidant activities. They performed HPLC analyses of flower water extracts and detected benzoic acid (161.68 mg/100 g DW), caffeic acid (100.11 mg/100 g DW), o-coumaric acid (42.09 mg/100 g DW), p-hydroxybenzoic acid (14.13 mg/100 g DW), ellagic acid (12.17 mg/100 g), naringenin (145.03 mg/100 g DW), quercetin (111.96 mg/100 g DW) and kaempferol (44.49 mg/100 g DW). In the current study, the methanolic leaf extracts contained quercetin in comparable amounts to those found previously in the flower water extracts. Dube et al. [43] studied five Southern Africa *Acacia* species (*A. karroo*, *A. nilotica*, *A. senegal*, *A. eriolaba* and *Faidherbia albida*) aceton:water (7:3, *v*/*v*) leaf extracts for their proanthocyanidin content; however they performed only general spectrophotometric assays, and they did not study *A. saligna*. *A. karroo* showed the highest and *A. senegal* the lowest phenolic compound contents based on Folin–Ciocalteu assay.

The methanolic leaf extracts of *L. inermis* growing in Northern Saudi Arabia investigated in the current study contained high amounts of apigetrin, at 1180.9 mg/100 g DW; apigenin 5-glucoside, at 596.3 mg/100 g DW; as well as gallic acid, at 81.0 mg/100 g DW (Table 1 and Figure 1B). Phenolic compounds, including flavonoids, tannins, coumarins, and naphthoquinones, are particularly abundant ingredients of *L. inermis* leaf extracts [22]. Mikhaeil et al. [44] studied the Egyptian *L. inermis* leaf extracts and detected *p*-coumaric acid from phenolic acids and, similar to our samples, apigenin and apigetrin (cosmosiin) from flavonoids. The apigenin derivative 4′-methoxyapigenin (linarigenin, linarisenin) was also detected by Mahmoud et al. in *L. inermis* leaf extracts of Egyptian origin [45]. Gallic acid was confirmed by other scientists from Iran [46]. Moreover, the presence of *p*-coumaric acids was reported by Mikhaeil et al. [44], but this compound was not detected in the Saudi Arabian henna leaf extract.

Leaf extracts of *A. saligna* and *L. inermis* showed strong antioxidant effects using different assays. *A. saligna* showed higher antioxidant effects compared to *L. inermis.* The higher antioxidant effects of *A. saligna* can mainly be attributed to the polyphenolic composition of leaves, including hyperoside, quercetin 3-glucuronide, gallic acid, *p*-coumaric acid, quercitrin and rutoside. These polyphenols showed strong antioxidant activities when examined individually. In agreement with the current study, a previous investigation on polyphenols showed that hyperoside has comparable antioxidant activities to other polyphenols, such as caffeic and ellagic acids [47]. Quercetin, as a flavonoid glycoside, is common in plants and in the human diet, reduces ROS production and may stimulate THP-1 acute monocytic leukemia in vitro [48]. Previous investigation found that quercetin has antioxidant and antiproliferative activities against RAW264.7 cancer Cells [49]. In the current study, we found strong quercetin 3-glucuronide antioxidant activities using different assays, and this is the first study exploring the antioxidant and biological activities of this quercetin derivative. Gallic acid (3,4,5-trihydroxybenzoic acid) is a known triphenolic compound that has strong antioxidant activities, as found in this study [50]. It was found that *p*-coumaric acid is a strong antioxidant, which is in agreement with previous investigations [51,52]. In *L. inermis*, moderate antioxidant activities were found, and these were attributed to the polyphenolic composition of leaf extracts including apigenin 5-glucoside. Apigenin has in vitro and in vivo antioxidant effects, as well as anti-mutagenic and anti-inflammatory effects, by inhibiting the cell cycle, inducing apoptosis and diminishing oxidative stress [53]. This is the first study to investigate the antioxidant and biological activities of the apigenin-derivative apigenin 5-glucoside and to show strong antioxidant activities.

Few studies have investigated the antioxidant activities of *Acacia* species; for example, Egyptian *A. saligna* flower water extracts showed weak antioxidant and antibacterial activities but had good antifungal activity against *Penicillium chrysogenum* using the disc diffusion method [18]. In another study, the Egyptian *Acacia nilotica* and *Acacia seyal* leaf extracts showed higher antioxidant activities than *Acacia laeta* extracts [19]. To our best knowledge, this is the first investigation on the antioxidant activities of Saudi *A. saligna.* In Tunisian *L. inermis*, the butanolic fraction showed strong antioxidant activities, and these activities were attributed to phenolic glycosides including 1,2,4-Trihydroxynaphthalene-1-O-β-d-glucopyranoside [23]. In another study, Tunisian *L. inermis* leaf and seed aqueous extracts showed antioxidant activities, but no active compounds were identified [24]. The Iranian *L. inermis* aqueous leaf extracts (Henna leaves) showed diverse antioxidant activities associated with ecotypes [25].

MTT and flow cytometry assays revealed that *A. saligna and L. inermis* leaf extracts have antiproliferative and cytotoxic activities against different cancer cells. These activities were attributed to the polyphenolic composition including rutoside, hyperoside, quercetin, quercetin 3-glucuronide and *p*-coumaric acid. Rutoside (rutin) has known antiproliferative and cytotoxic activities against different cancer cells [54]. A previous investigation showed that hyperoside has anticancer activity against lung cancer cells by upregulating FoxO1 via CCAT1 [55]; additionally, it showed an inhibitory effect against SW620 human colorectal cancer cells via the induction of the p.53 signaling pathway and apoptosis [56]. Apigenin (4′,5,7-trihydroxyflavone) is a common component of the human diet, especially in fruits, vegetables and herbs, with known antiproliferative and proapoptotic bioactivity [57]. The current study is the first to report on the antiproliferative and cytotoxic activities of the apigenin derivative apigenin 5-glucoside.

Gallic acid showed strong antiproliferative activities against the different cancer cells examined in this study, which is in agreement with previous investigations [50]. Gallic acid obtained from mango peels has antiproliferative effects against human colon adenocarcinoma cells and mouse connective cells, which can be attributed to its antioxidant activities [50]. Quercetin is recognized as an antiproliferative factor against specific cancer cells and has been shown to reduce the activity of specificity protein 1 and minimize the proliferation of human carcinoma HepG2 cells [57,58]. Quercetin has antiproliferative activities against prostate cancer cells, which can be attributed to its synergy with other polyphenols in green tea [58]. In addition, quercetin has cytotoxic activity against lung cancer cells [59]. In the current study, quercetin 3-glucuronide (quercetin derivative) was shown for the first time to exhibit antiproliferative and cytotoxic activities against different cancer cells. Cytotoxic spirostane saponin and biflavonoid glycoside were identified in the leaves of Egyptian *A. saligna* and showed anticancer activities against HepG2 cancer cells [42]. A previous investigation on the aerial parts of Acacia species *(A. salicina*, *A. laeta*, *A. hamulosa*, and *A. tortilis)* from the eastern region of Saudi Arabia (approximately 700–1000 km from the Riyadh region) revealed that *A. laeta* and *A. hamulosa* have cytotoxic activities against HepG2 and breast cancer cell line [20]. In a previous investigation, leaf aqueous and methanolic extracts of *Indian* *L. inermis* showed significant antioxidant activities that inhibited Cr (VI)-induced cytotoxicity in MDA-MB-435S breast cancer cells, and these activities were associated with phenolic compounds including gallic acid and p-hydroxybenzoic acid [26].

*A. saligna* and *L. inermis* leaf extracts had antibacterial effects against most bacteria investigated. *A. saligna* exhibited two-fold higher antibacterial activities than *L. inermis* against all bacteria. The most sensitive bacteria were *E. coli* and *S. aureus*. Identified polyphenols (rutoside, apigenin 5-glucoside and *p*-coumaric acid) showed comparable antibacterial activities to the standards. A previous investigation on Egyptian *A. saligna* showed that flower water extracts had weak antibacterial activities but good antifungal activity against *Penicillium chrysogenum* using the disc diffusion method [18]. In another investigation, *A. saligna* from Egyptian methanolic leaf extract exhibited no antibacterial activities against most bacteria examined [17]. Another study on the aerial parts of other *Acacia* species revealed that *A. laeta* and *A. tortilis* have antimicrobial activities against *S. aureus*, *E. coli*, and *P. aeruginosa* [20]. Tunisian *L. inermis* leaf and seed aqueous extracts showed antimicrobial activities, but no active compounds were identified [24]. Iranian *L. inermis* aqueous leaf extracts showed strong antibacterial activities against *S. aureus*, *Streptococcus agalactiae*, *B. cereus*, *Corynebacterium pseudotuberculosis*, *Klebsiella pneumonia*, *E. coli* and *Salmonella enterica* serovar *typhi* [25]. However, no active compounds were identified. The antibacterial activities of *A. saligna and L. inermis* found in this study are attributed to the moderate to high contents of polyphenols such as rutoside, apigenin 5-glucoside and *p*-coumaric acid. Rutoside (rutin) has known antibacterial activities against *E. coli*, *Proteus vulgaris*, *Shigella sonnei*, *P. auruginosssa* and *B. subtilis* [54]. Apigenins such as aglycone have antibacterial activity against *E. coli* and *P. aeruginosa* [60]; however, this is the first report on the wide-spectrum biological activities of apigenin 5-glucoside. *P-*coumaric acid exhibited antibacterial activity against *B. cereus* and *Salmonella typhimurium* when applied with niacin [61]. In the current study, we found moderate antibacterial activities of hyperoside, quercetin and quercetin 3-glucuronide. Quercetin showed moderate antibacterial activities against *S. aureusm*, *E. coli* and *P. aeruginosa* in previous investigations [62,63].

*A. saligna and L. inermis* extracts showed strong antifungal effects against most fungi studied (Table 3). *A. saligna* showed two to three-fold higher activity (lower IC_50_ values) than *L. inermis.* Rutoside, hyperoside, apigenin 5-glucoside and *p*-coumaric acid showed the highest antifungal effects among the studied polyphenols.

*A. saligna* and *L. inermis* extracts showed strong antifungal effects against the studied fungi. *A. saligna* showed two to three-fold higher activity than *L. inermis.* The identified polyphenols including rutoside, hyperoside, apigenin 5-glucoside and *p*-coumaric acid showed the highest antifungal activities, explaining the activities of leaf extracts. A previous investigation on Egyptian *A. saligna* showed that flower water extracts had good antifungal activity against *Penicillium chrysogenum* using the disc diffusion method [18]. A study on the aerial parts of other *Acacia* species revealed that *A. laeta* and *A. tortilis* have antifungal activities against *C. albicans* [20]. Chinese *Acacia confuse* seeds contained a protein showing strong antifungal activities against *Rhizoctonia solani* [64]. Apigenin 5-glucoside showed strong antifungal activities against *C. albicans* in the current study, which were comparable or greater than those of apigenin and its derivatives found previously [65]. These antifungal activities were attributed to the reduction of intra and extracellular reactive oxidative species as a mechanism of antifungal action. The current study is the first comprehensive illustration of the fungicidal activities of the apigenin-derivative apigenin 5-glucoside. Rutoside has antifungal activities against *C. albicans* [54]. A previous investigation showed that hyperoside had antifungal activities against *Alternaria alternata*, *Pestalotia guepinii*, *Fusarium avenaceum*, *Drechslera* sp. and *Epicoccum nigrum* [66]. *P-*coumaric acid showed antifungal effects against *Botrytis cinerea* [67]. Quercetin has antifungal activities against *C. albicans* [68,69,70]. However, this is the first report on the antifungal activities of apigenin 5-glucoside.

## 5. Conclusions

To the best of our knowledge, this is the first study to explore the polyphenol composition and biological activities of methanolic leaf extracts of natural *A. saligna* and *L inermis* populations from northern Saudi Arabia. Several polyphenols were tentatively identified in the leaf extracts including rutoside, hyperoside, gallic acid, quercetin 3-glucuronide and *p*-coumaric acid in *A. saligna* and apigetrin, apigenin 5-glucoside and gallic acid in *L. inermis*. Indeed, further analyses are needed to better understand the complete composition of the studied plants. The MTT and flow cytometry assays revealed that *A. saligna* and *L. inermis* leaf extracts had antiproliferative and cytotoxic activities against different cancer cells. These activities were attributed to the polyphenolic composition, including rutoside, hyperoside, quercetin, quercetin 3-glucuronide and *p*-coumaric acid, and necrotic cell accumulation during apoptotic periods. Leaf extracts of *A. saligna* and *L. inermis* showed strong antioxidant effects using different assays. *A. saligna* showed higher antioxidant effects compared to *L. inermis.* The higher antioxidant effects of *A. saligna* can mainly be attributed to the polyphenolic composition of leaves, including hyperoside, quercetin 3-glucuronide, gallic acid, *p*-coumaric acid, quercitrin and rutoside. *A. saligna and L. inermis* leaf extracts had antibacterial effects against most bacteria investigated. *A. saligna* had twice higher antibacterial activities than *L. inermis* against all bacteria. The most sensitive bacteria were *E. coli* and *S. aureus*. The identified polyphenols (rutoside, apigenin 5-glucoside, and *p*-coumaric acid) showed comparable antibacterial activities to the standards. Both species exhibited antifungal activities, which were attributed to their polyphenol composition and associated with specific polyphenols including rutoside, hyperoside, apigenin 5-glucoside and *p*-coumaric acid. The current study is the first to report on the antiproliferative, cytotoxic, antibacterial and antifungal activities of the apigenin-derivative apigenin 5-glucoside.

## Figures and Tables

**Table 1:** Polyphenol contents of *A. saligna* and *L. inermis* leaf extracts (mg/100 g dry weight (DW) ± SD).

Compounds		*Acacia saligna*	*Lawsonia inermis*
**Phenolic acids**	Gallic acid	136.2 ± 11.1	81.0 ± 13.2
	*p*-Coumaric acid	34.8 ± 2.9	nd *
**Flavonoids**	Hyperoside	632.5 ± 45.5	nd
	Isoquercetin	73.2 ± 9.2	nd
	Quercetin	6.1 ± 0.5	nd
	Quercetin 3-glucuronide	125.5 ± 2.5	nd
	Rutoside	1533.0 ± 55.7	nd
	Apigetrin	nd	1180.9 ± 153.3
	Apigenin 5-glucoside	nd	596.3 ± 49.1

Values are expressed as mean ± standard deviation (SD). nd * not detected.

**Table 2:** Antibacterial effects of *A. saligna* and *L. inermis* methanolic extracts, rutoside, hyperoside and apigenin 5-glucoside, *p*-coumaric acid, quercetin and quercetin 3-glucuronide by means of minimum inhibitory (MIC) and bactericidal concentration (MBC) in mg/mL. Values are the means of three replicates.

	*B. cereus* MIC MBC	*P. aeruginosa* MIC MBC	*L. monocytogenes* MIC MBC	*E. coli* MIC MBC	*M. flavus* MIC MBC	*S. aureus* MIC MBC
***A. saligna***	0.35 ± 0.01	0.37 ± 0.02	0.47 ± 0.03	0.31 ± 0.03	0.41 ± 0.02	0.30 ± 0.05
	0.73 ± 0.03	0.79 ± 0.03	0.99 ± 0.05	0.72 ± 0.01	0.85 ± 0.03	0.73 ± 0.03
***L. inermis***	0.43 ± 0.03	0.41 ± 0.03	0.42 ± 0.03	0.34 ± 0.02	0.52 ± 0.05	0.41 ± 0.02
	0.86 ± 0.04	0.96 ± 0.05	0.93 ± 0.02	0.75 ± 0.03	1.53 ± 0.12	0.93 ± 0.03
**Rutoside**	0.11 ± 0.01	0.07 ± 0.01	0.11 ± 0.01	0.12 ± 0.01	0.12 ± 0.01	0.11 ± 0.01
	0.22 ± 0.01	0.12 ± 0.01	0.21 ± 0.02	0.23 ± 0.01	0.23 ± 0.01	0.25 ± 0.03
**Hyperoside**	23.3 ± 2.4	27.2 ± 3.53	34.3 ± 4.21	31.2 ± 1.43	22.42 ± 1.55	19.54 ± 2.41
	>500	>500	>500	>500	>500	>500
**Apigenin 5-glucoside**	0.13 ± 0.01	0.12 ± 0.01	0.10 ± 0.01	0.09 ± 0.01	0.10 ± 0.01	0.13 ± 0.02
	0.33 ± 0.03	0.31 ± 0.03	0.28 ± 0.02	0.27 ± 0.03	0.30 ± 0.03	0.33 ± 0.03
***p*-Coumaric acid**	0.12 ± 0.01	0.06 ± 0.01	0.26 ± 0.02	0.12 ± 0.01	0.16 ± 0.02	0.23± 0.02
	0.31 ± 0.03	0.22 ± 0.02	0.58 ± 0.03	0.25 ± 0.02	0.38 ± 0.03	0.47 ± 0.03
**Quercetin**	30.6 ± 3.1	31.8 ± 2.11	43.7 ± 3.51	38.6 ± 3.14	28.65 ± 2.11	21.53 ± 1.53
	>500	>500	>500	>500	>500	>500
**Quercetin 3-glucuronide**	35.2 ± 3.1	25.3 ± 1.54	32.1 ± 3.78	31.8 ± 3.32	21.65 ± 2.11	17.1 ± 1.53
	>500	315.14 ± 18.42	>500	>500	411.17 ± 22.67	427 ± 27.23
**Streptomycin**	0.07 ± 0.01	0.11 ± 0.01	0.12 ± 0.01	0.11 ± 0.01	0.10 ± 0.01	0.15 ± 0.01
	0.16 ± 0.02	0.21 ± 0.02	0.21 ± 0.02	0.20 ± 0.01	0.21 ± 0.02	0.31 ± 0.02

**Table 3:** Minimum inhibitory (MIC) and fungicidal concentration (MFC) of *A. saligna* and *L. inermis* methanolic extracts, rutoside, hyperoside, apigenin 5-glucoside, quercetin, quercetin 3-glucuronide and *p*-coumaric acid. Values are means of three replicates in mg/mL.

	*Aspergillus flavus* MIC MFC	*Aspergillus ochraceus* MIC MFC	*Aspergillus niger* MIC MFC	*Candida albicans* MIC MFC	*Penicillium funiculosum* MIC MFC	*Penicillium ochrochloron* MIC MFC
***A. saligna***	0.30 ± 0.02	0.38 ± 0.02	0.48 ± 0.03	0.58 ± 0.03	0.43 ± 0.03	0.44 ± 0.05
	0.91 ± 0.05	0.95 ± 0.3	1.02 ± 0.05	1.42 ± 0.18	1.01 ± 0.06	1.31 ± 0.12
***L. inermis***	0.45 ± 0.05	0.47 ± 0.03	0.64 ± 0.05	0.88 ± 0.04	0.75 ± 0.02	0.79 ± 0.03
	0.98 ± 0.06	1.07 ± 0.05	1.45 ± 0.17	1.86 ± 0.13	1.53 ± 0.05	1.64 ± 0.12
**Rutoside**	0.21 ± 0.02	0.18 ± 0.01	0.28 ± 0.03	0.25 ± 0.03	0.30 ± 0.02	0.23 ± 0.03
	0.45 ± 0.03	0.55 ± 0.03	0.62 ± 0.03	0.51 ± 0.03	0.71 ± 0.03	0.43 ± 0.03
**Hyperoside**	0.10 ± 0.02	0.13 ± 0.03	0.15 ± 0.03	0.21 ± 0.03	0.25 ± 0.03	0.31 ± 0.03
	0.46 ± 0.03	0.50 ± 0.05	0.52 ± 0.04	0.93 ± 0.07	1.03 ± 0.11	1.19 ± 0.10
**Apigenin 5-glucoside**	0.11 ± 0.03	0.17 ± 0.02	0.23 ± 0.03	0.03 ± 0.01	0.75 ± 0.05	0.59 ± 0.05
	0.84 ± 0.04	1.03 ± 0.08	1.11 ± 0.15	0.08 ± 0.01	1.33 ± 0.14	1.26 ± 0.13
**Quercetin**	0.31 ± 0.03	0.20 ± 0.02	0.21 ± 0.01	0.06 ± 0.01	0.24 ± 0.03	0.29 ± 0.03
	0.63 ± 0.05	0.75 ± 0.03	0.75 ± 0.02	0.33 ± 0.03	0.70 ± 0.04	0.63 ± 0.03
**Quercetin 3-glucuronide**	0.26 ± 0.03	0.17 ± 0.03	0.18 ± 0.02	0.06 ± 0.01	0.21 ± 0.01	0.26 ± 0.02
	0.52 ± 0.03	0.61 ± 0.05	0.62 ± 0.05	0.27 ± 0.03	0.60 ± 0.05	0.54 ± 0.05
***p*-Coumaric acid**	0.22 ± 0.03	0.23 ± 0.02	0.21 ± 0.01	0.32 ± 0.03	0.22 ± 0.01	0.20 ± 0.02
	0.43 ± 0.01	0.45 ± 0.05	0.41 ± 0.03	0.60 ± 0.01	0.59 ± 0.05	0.40 ± 0.03
**KTZ (Ketoconazole)**	0.20 ± 0.01	0.23 ± 0.02	0.10 ± 0.01	0.22 ± 0.01	2.05 ± 0.13	0.21 ± 0.01
	0.41 ± 0.03	0.46 ± 0.03	0.21 ± 0.03	0.43 ± 0.02	3.51 ± 0.11	0.43 ± 0.05

## Supplementary Materials

The following are available online at https://www.mdpi.com/2223-7747/9/7/908/s1.
