# Supplementary figures and images for: Antioxidant and Biological Activities of Acacia saligna and Lawsonia inermis Natural Populations
Source: Plants (Basel). 2020 Jul 17;9(7):908. doi: 10.3390/plants9070908 (PMC7411707; doi:10.3390/plants9070908)

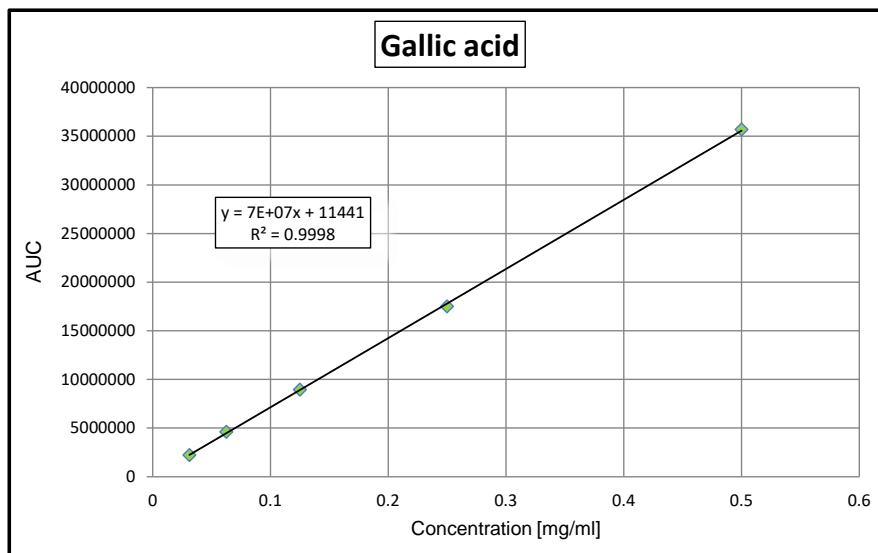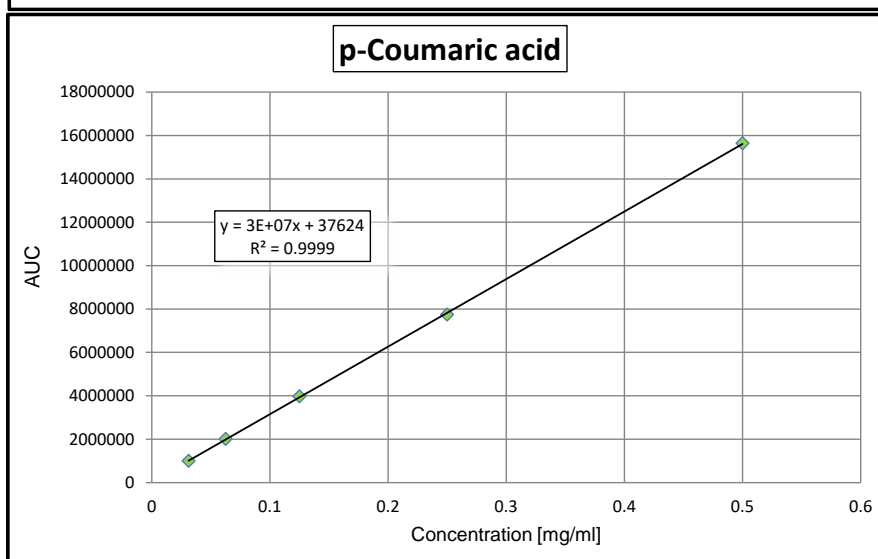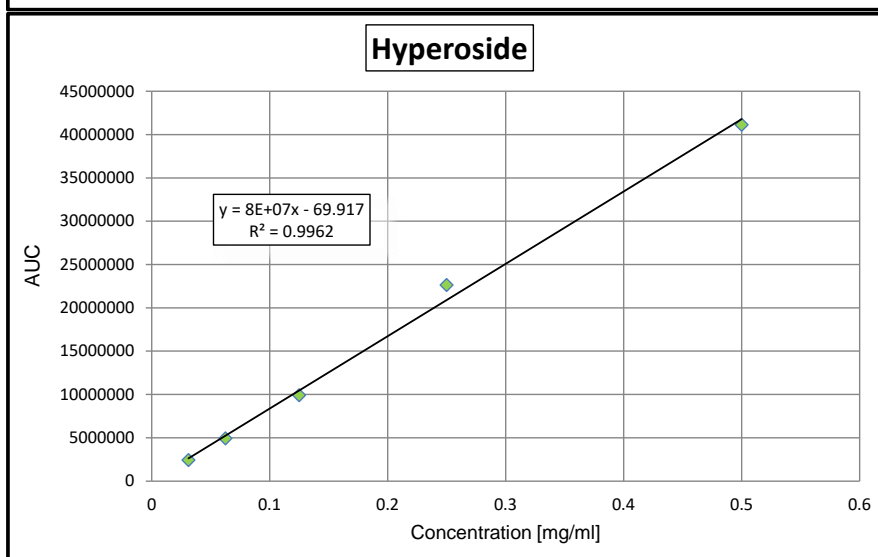

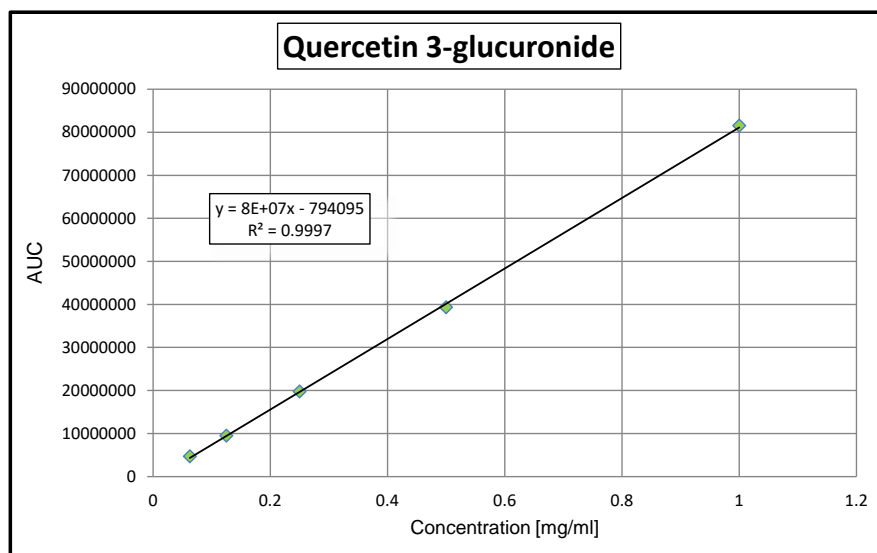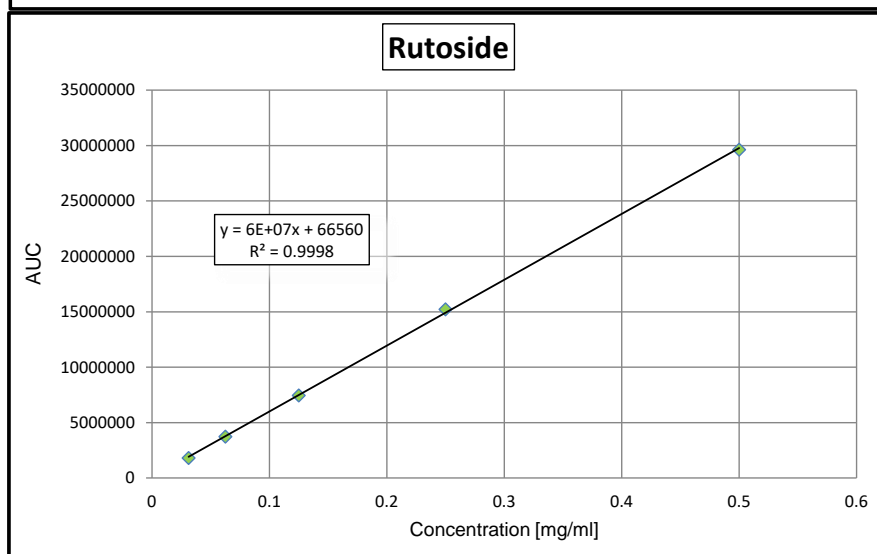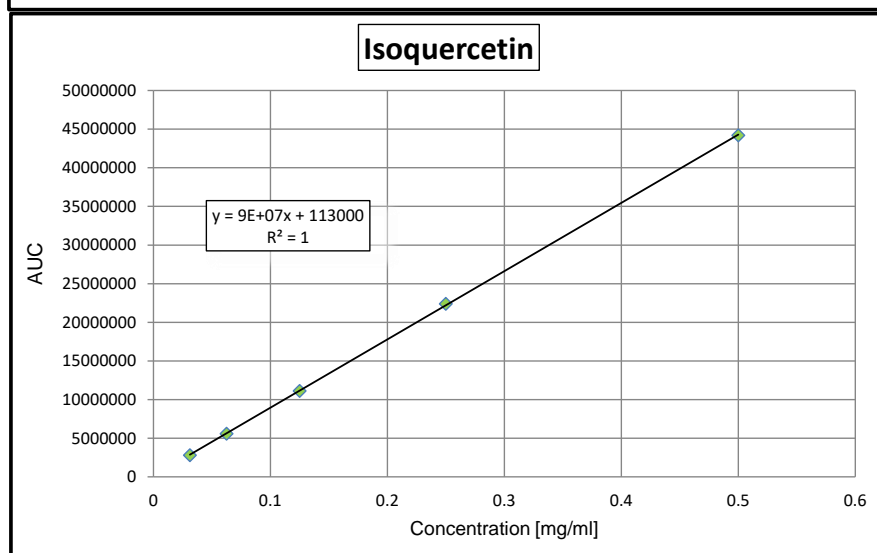

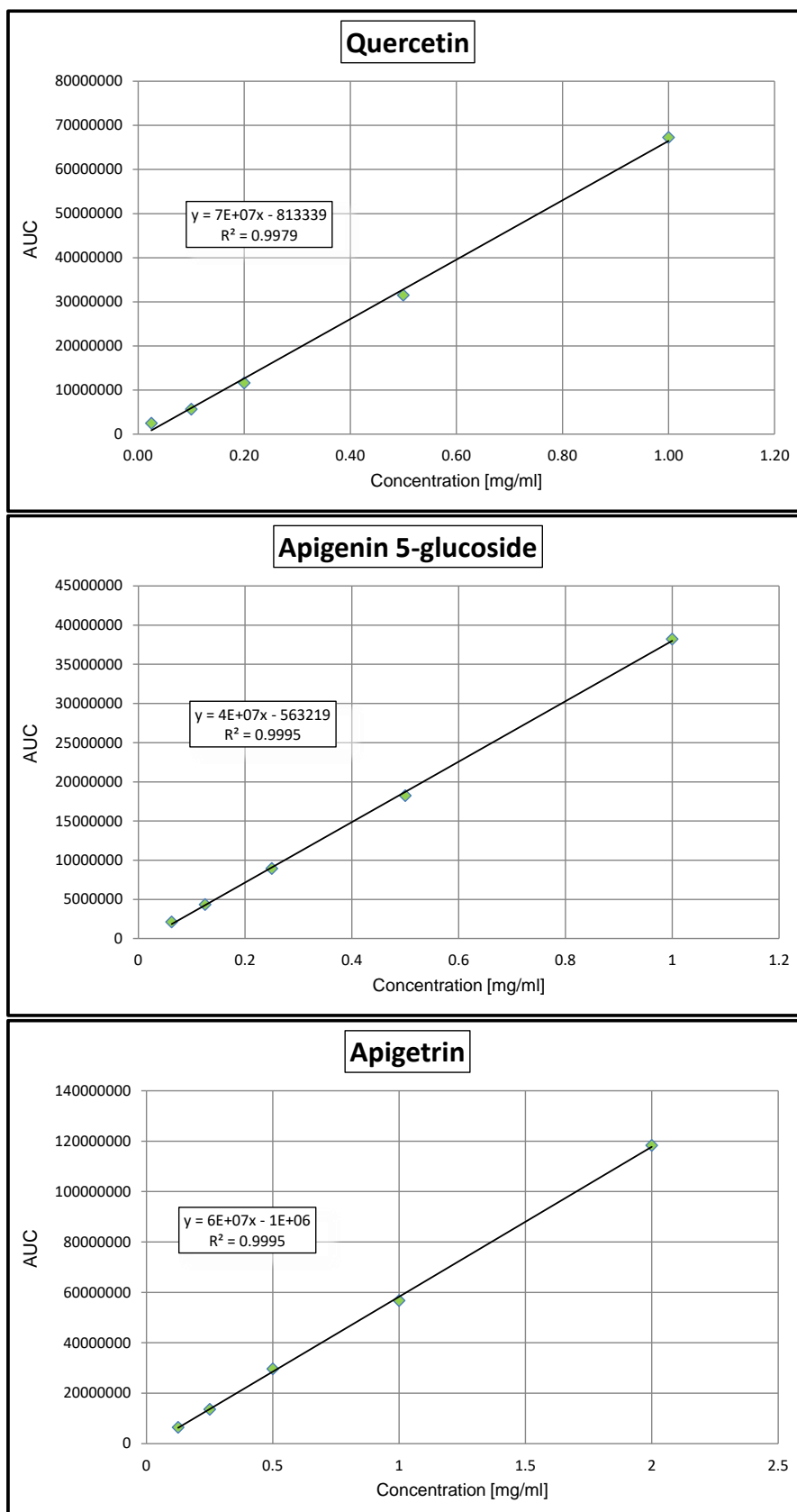

**Figure S1.** The calibration curves of detected compounds.

Supplement: Supplementary file 1 [file plants-09-00908-s001.pdf]
